# Erysipelas of the Larynx

**Published:** 1886-08

**Authors:** 


### *ERYSIPELAS OF THE LARYNX.*

M. F. Massie, in *Il Morgagui*, publishes his observations on erysipelas of the larynx, and is continuing his studies of the subject by inoculating the streptoeoccus of erysipelas. At the conclusion of his paper, he summarizes the following facts:

1. There is such an affection as primary erysipelas of the larynx.

2. A great number of cases described as œdema of the larynx, are really erysipelas.

3. There are two forms of laryngeal erysipelas: One in which the local affection predominates, and the other in which the constitutional symptoms are most marked.

4. The method of treatment consists: (*a*), Abstraction of heat and revulsives; (*b*), scarification, and, if this is not sufficient, (*c*), tracheotomy.

